# Policy differentiation and the politics of belonging in India’s emigrant and emigration policies

**DOI:** 10.1080/13621025.2023.2244451

**Published:** 2023-08-08

**Authors:** Mira Burmeister-Rudolph

**Affiliations:** Department of Political Science, University of Amsterdam, Amsterdam, The Netherlands

**Keywords:** Citizenship, Emigrant policy, Emigration, India, Rights, Post-colonial Legacies

## Abstract

India is the largest emigrant origin country in the world. The majority of Indian emigrants work in low-wage employment in the Gulf Cooperation Council countries. This paper contrasts India’s policy responses directed at migrants living in the Gulf countries with policies targeted at those in the Global North. India has extended substantive rights and symbolic inclusion to Indian citizens and (descendants of) former citizens residing in the Global North. However, while it established a social protection framework for low-wage emigrants in the Gulf, these emigrants are often unable to access other substantive citizenship rights and are mostly ignored at the symbolic level. Through critical approaches to the study of diasporas and the lens of boundary work, this article analyzes how emigrant origin states (re-)define belonging through differentiated emigration and emigrant policies. It shows that the Indian state links inclusive/exclusive boundaries of (symbolic) national membership, inherent in emigration and emigrant policies, to classifications regarding emigrants’ social identities, in this case class and religion.

## Introduction

Postcolonial studies on citizenship (e.g. Chung 2017; Kabeer 2006) note that legal citizenship status does not always equate to rights and belonging (Yuval-Davis 2006); they highlight its exclusionary dimensions, such as gender, class, and race. Legal citizenship, in an extreme case, can merely be a status of citizenship-as-nationality (Liu 2022). On the other hand, considerable research (e.g. Délano and Gamlen 2014; Levitt and de la Dehesa 2003; Waterbury 2010) emphasized that citizenship has increasingly been de-territorialized (Bloemraad 2004; Vertovec 2009), and that emigrant states provide former citizens and their kin with citizenship-like rights which signify membership of a state.

If the provision of rights is independent of formal citizenship status, what explains their differentiated distribution in emigrant policies? In this article, I focus on the case of India’s differentiated emigrant and emigration policies. India is the largest migrant origin country globally and has been for several years (IOM 2021). The Gulf Cooperation Council (GCC) countries have been a key destination for Indian labor migrants since the 1970s, and host an estimated 9.6 million out of 17.9 million Indian citizens residing abroad (UN DESA 2021). Indian workers emigrating to the GCC countries are mainly low-wage^1^ workers (Khadria 2006).^2^ As in other cases of South-South temporary contract migration, the experience of protracted precarity is central to the migration process (Piper and Withers 2018). On paper, India has established an institutional social protection framework for low-wage labor emigrants, e.g. an insurance scheme and emergency funding (see Appendix Table 1).

The Indian state has also made intensive political efforts to extend symbolic and substantive rights and privileges to Indian citizens and naturalized former citizens residing in North America, Europe, and Australasia (Lall 2003; Xavier 2011), who often work in high-skilled professions (OECD n.d.). Consequently, non-citizens of Indian descent and Indian citizens (abroad and at home) enjoy almost equal citizenship rights (see Appendix Table 4). However, low-wage labor migrants often cannot access substantive citizenship rights such as political rights, e.g. participation in elections abroad. Indian emigrant policies – the formal rules that define the obligations and rights of absent citizens, persons of Indian descent, and kin members (see Pedroza 2020) – are thus not unitary but differentiated. This differentiation also holds for India’s emigration policies, which are the formal rules administrating the exit of citizens (Pedroza 2020). The Indian state regulates and controls the emigration of low-wage migrants, whereas emigrants bound to countries of the Global North are not subject to exit restrictions (Rajan, Varghese, and Jayakumar 2010).

By contrasting India’s policy responses directed at its citizens living abroad and former citizens and their descendants, I examine how India (re)-configures belonging. I build on a conceptual framework of citizenship consisting of identity, rights, and status dimensions (Joppke 2007) and view the different levels of rights and entitlements enshrined in emigration and emigrant policies as an outcome of the politics of belonging, in which ‘specific political projects (aim) at constructing belonging in particular ways to particular collectives (…)’ (Yuval-Davis 2006, 197). Citizenship as identity – here the normative conceptions which underpin membership boundaries applied by the state (see Joppke 2007) – has consequences for how rights and privileges are redistributed. Applied to emigrant policies, the notion of politics of belonging echoes constructivist, critical approaches to diasporas, in which stakeholders construct the belonging of emigrants to a political community (see Burgess 2020; Ho 2011). It views diasporas ‘as imaginations of communities which unite segments of people that live in territorially separated locations’ (Sökefeld 2006, 267), instead of conceptualizing diasporas as bounded, natural entities. The transformation of emigrants into a diaspora is grounded in ‘ideas that define migrants as members of a transnational community or relationships as relations of belonging’ (Sökefeld 2006, 270). Policies aiming to establish an emigrant-origin state relationship act as the means to materialize these ideas of transnational belonging (Bauböck 2010). However, emigrant policies are selective. Emigrant states privilege specific attributes and identities – often professionals and business class emigrants (Ho 2011) – and can reinforce essentialist (national) identities, while neglecting others who do not fit into their narratives of diaspora (see Ho 2011), thus creating ‘absent spaces of citizenship’ (Ho 2011, 768) for the latter.

Through the lens of boundary work, this research scrutinizes how changing notions of inclusion and exclusion form membership definitions and belonging, by means of emigrant and emigration policies. The analytical framework of boundary work gives insights into how institutionalized social difference is produced, organized, and contested – the ‘axes of neglect’ (Ho 2011, 764), consisting of absences from state definitions of diaspora and belonging. This study demonstrates that the differentiation in the emigrant and emigration policy of origin states results, in the case of India, from the state’s perception of different groups of citizens. Importantly, I show that the Indian state links inclusive/exclusive boundaries of (symbolic) national membership to classifications of emigrants’ social identities, in this case class and religion.

This article aims to complement existing research, which has focused predominantly on boundary work in immigration and integration policy, by bringing in emigration states and foregrounding citizenship as a central site of analysis. Emigration states, like immigration states, categorize migrants’ value and deservingness using social classifications, such as class and religion. For the former, however, boundary work is not reflected in bordering practices to demarcate and exclude non-citizens from citizenship; rather, it is designed to include non-citizens while differentiating among citizens. It is thus necessary to scrutinize closely how boundary making plays out in emigration and emigrant policies. This is in line with approaches to study boundaries not as binary us-them constructions, but as multivalent, constituted (Yurdakul and Korteweg 2021) and linked to the multiplicity of socio-spatial scales (see Amelina 2012), i.e. an emigrant’s citizenry and social-economic and political incorporation, at sub-national, national and international levels. In the case of India’s emigration and emigrant policies, multiple processes of boundary making are at work. These include boundary shifting and expansion through de-territorialized forms of membership and distribution of associated citizen rights, as well as, simultaneously, the consolidation of social boundaries along religious and classed lines through mostly symbolic boundary acts, and the contracting of citizenship boundaries, via, for example, the National Register of Citizens (see Punathil 2022).

India is a relevant case to study, because of its total emigrant numbers and particularly due to the colonial legacies inherent in its emigrant and emigration policies, which continue to inform contestations over the content and boundary lines of membership. The Indian case can serve as a starting point for analyzing other postcolonial migration states (Sadiq and Tsourapas 2021) with migration corridors to the GCC and Global North countries for both low and high-wage employment, such as the Philippines, Bangladesh, Egypt, Ghana, and Kenya; such analysis can further explore why certain countries show higher levels of differentiation in their emigration and emigrant policies than others. Overall, India shows a stark differentiation in its policies compared to other countries: differentiation in the Philippines, Sri Lanka, and Nepal is limited to emigration regulations, with temporary travel bans exclusively targeting female domestic workers to the GCC countries (Shivakoti, Henderson, and Withers 2021). While Egypt has differentiated emigrant policies depending on migrants’ skill levels and migration duration, it does not impose any exit restrictions on its nationals (see Tsourapas 2015).

The outline of the paper is as follows. First, I discuss emigrant and emigration policies as sites of stratification-generating social and symbolic boundaries, of in- and exclusion of membership of the nation(−state). Next, to contextualize the study, I provide an overview of India’s emigration history and consider the marginalization of low-wage labor migrants in emigrant and emigration policies in context, as a continuation of their overall precarious status. Finally, in the empirical section, I scrutinize how emigration and emigrant policies distribute rights and privileges, not based on emigrants’ citizenship status, but on religious and classed classifications. I consider, first, the Emigration Act (1983), which created categories of low- and high-skilled migrants, and subsequently informed all Indian emigrant policies. Second, I examine the institutionalization of India’s relationship with its migrants, through the establishment of the Ministry of Overseas Indian Affairs (MOIA) in 2004 and its emigrant policies: voting rights in the context of forms of Indian citizenship, and the Overseas Indian Day and Award.

### The selectiveness of emigrant and emigration policies, and these policies as sites of symbolical and substantive in/exclusion

Categorizations used as selection criteria are a vital tool for governing (migrant) populations and mobility (see Welfens 2021). A plethora of literature has shed light on boundary work in the immigration context, exploring how states classify people as belonging to ‘us’ or ‘them’ through selective visa policies, rights attribution, and pathways to citizenship. As boundary work concerns the construction of identity in relation to others, research adopting this approach, with a focus on bureaucratic boundary drawing in immigration contexts, has scrutinized boundaries as institutional regulations which draw distinctions between migrants and refugees as non-citizens, as opposed to citizens (e.g. Anderson 2013; Raghuram 2021; Welfens 2021). Boundary making, of course, is not unique to immigration contexts: States also select among their emigrants to restrict exit (see Shivakoti, Henderson, and Withers 2021) and the distribution of rights and privileges (Ho 2011; Mylonas and Žilović 2019; Pedroza 2020; Tsourapas 2015). They do so based on gendered and classed criteria and category attribution, e.g. skill-level or destination country.

Central to boundary making is subject-making, which applies in a similar way to how states govern their citizens and non-citizens. As Anderson (2013, 4) has pointed out, ‘the community of value is defined from the outside by non-citizens, but also from the inside, by the “Failed Citizen”’. Anderson (2013) explores citizenship in its relation to ‘communities of value’, arguing that nation-states, instead of emphasizing common legal status to bind people together, portray national membership as grounded in shared values, such as common ideals and patterns of social behavior, for example, economic worth, self-sufficiency, and progressiveness. ‘Failed Citizens’ are thus symbolically excluded from the national political community, as they are viewed as lacking value and values. On the other hand, the community of value includes high-skilled labor immigrants, independent of their citizenship status (Anderson 2013).

Similarly, as Agarwala (2022), Dickinson and Bailey (2007), Ho (2011), and Varadarajan (2010) have demonstrated, middle-classness and professional success are decisive values for the recognition of emigrants in emigrant policies. Importantly for this study, Anderson’s body of work also laid the ground for scholarship which has moved beyond examining the conditions of membership along ethnic boundaries (Wimmer 2008), but instead foregrounds the multi-dimensionality of inequality as a result of intersectional classifications (Amelina 2012; Bonjour and Duyvendak 2018; Fernandez 2021; Yurdakul and Korteweg 2021). Fernandez (2021) describes the kafala in the GCC countries as a system of racialized governance of labor migration, which creates hierarchies among immigrants based on race, gender, nationality, and migration status, and in which South Asian persons in low-wage employment rank at the bottom of the hierarchy.

Policies exclude individuals and communities as target groups (Mylonas and Žilović 2019). Emigrant and emigration policies are thus the outcome of ‘how the state (…) classifies those who move across its borders in different groups, and, based on that (…) which rights these persons have according to the groups that they fall into’ (Pedroza 2020, 321). To understand how states categorize emigrants into different groups through emigration and emigrant policies, and how these policies create hierarchical orders of national belonging and membership, I turn to the concept of boundary work (see Amelina 2012; Lamont and Molnár 2002; Wimmer 2008; Yurdakul and Korteweg 2021). Boundary work as an analytical framework provides insights into how social differences, manifested in classifications such as nationality, gender, class, race, ethnicity, and caste, are symbolically and substantively produced and organized. These institutionalized social differences result in ‘unequal access to and unequal distribution of resources (material and nonmaterial) and social opportunities’ (Lamont and Molnár 2002, 168). Boundary making aims to demarcate and regulate subjects (Yurdakul and Korteweg 2021), and social classifications structure these processes. I will show in the empirical section how, as a result of selective categorization practices, emigrant states (re)-produce hierarchies and social inequalities among emigrants through their policies.

According to Lamont and Molnár (2002), boundary making can preserve, enforce, normalize, rationalize, and contest institutionalized social differences. Yurdakul and Korteweg (2021) describe these strategies as material and symbolic registers of boundary-making. Symbolic boundaries are ‘conceptual distinctions made by social actors to categorize objects, people, practices and even time and space. […] (They) also separate people into groups and generate feelings of similarity and group membership’ (Lamont and Molnár 2002, 168). They operate as discursive constructions, which create notions of ‘inside(r)’ and ‘outside(r)’, traceable in texts and speech, for example, in policy briefs and parliamentary debates (Yurdakul and Korteweg 2021). Material boundaries are regulatory practices, such as the forming and implementation of laws and policies (Yurdakul and Korteweg 2021). Symbolic boundaries can turn into material boundaries but do not necessarily have to (Lamont and Molnár 2002; Yurdakul and Korteweg 2021).

Boundaries are not static, and literature has described various strategies of boundary (re)making – for example, shifting and modifying boundaries (Wimmer 2008) by crossing, blurring, expanding, contracting, and re-positioning (Wimmer 2008; Zolberg and Woon 1999). Emigrant policies exemplify the symbolic and material shifting of boundaries by expansion (see Wimmer 2008): Origin states utilize emigrant policies to designate the inclusion of emigrants in the nation-(state). Emigrant policies, such as migrant days, which celebrate emigrants’ achievements (Délano and Gamlen 2014), or the institutionalization of formal diaspora state offices (Gamlen 2019) are ‘embracing’ (Délano and Gamlen 2014) strategies of boundary work which focus on the production of ‘relationships of communication’ (Waterbury 2010, 142) and diasporic subjectivities (Raj 2015). They use (national) symbols to promote emigrants’ sense of enduring belonging (Levitt and de la Dehesa 2003; Waterbury 2010).

De-territorialized membership signifies belonging to a political community (Bauböck 2010; Naujoks 2020) and engages emigrants and former citizens in nation-building processes (Waterbury 2010). They are prime examples of more inclusive material boundaries resulting from legal amendments to citizenship definitions. These can be forms of ‘external citizenship’ for citizens who are long-term or permanent residents abroad (Bauböck 2009); the institutional transformation of legal membership by extending legal rights to individuals, resulting in multiple nationalities (dual citizenship) (Bauböck 2010); or extra-territorial citizenship, the partial provision of citizenship rights to non-nationals (Naujoks 2020). States that offer de-territorialized membership do not always provide all citizenship rights outside their territory (Naujoks 2020). Overseas Citizenship of India (OCI), for example, excludes political rights, such as voting, and does not have the same interstate functions as a passport. It does not, therefore, provide diplomatic protection and cannot be used as a means of international travel (see Faist, Gerdes, and Rieple 2004), but still provides certain privileges, such as visa-free entry and permission to work. Full Indian citizenship and the OCI can be situated on a continuum of citizenship rights, which I understand as a ‘layered’ citizenship regime, where different categories of citizenship with different levels of rights and entitlements co-exist (Mitra 2008).

Emigrants and (former) citizens actively shape emigrant and emigration policies by lobbying for and contesting them, thus engaging in practices of modifying the boundaries of Indian citizenship. For example, Xavier (2011) and Mani and Varadarajan (2005) suggest that the Indian state introduced the OCI card to accommodate the demands of the organized and activist groups of Indians abroad, predominantly those living in Europe and North America, who also lobbied for the establishment of the MOIA (Gamlen 2019). Low-wage labor migrants in the South Indian state of Kerala, and the Keralan state, were at the forefront of demanding the liberalization of emigration restrictions, eventually leading the Indian state to introduce the Emigration Act in 1983 (Agarwala 2022). Williams’ (2019) analysis of everyday encounters of OCI card applicants with the Indian emigration state, often marked by frustration and anxiety about the process, demonstrates that emigrant policies do not necessarily lead to a sense of belonging, but instead create emotional distance from the Indian state. These insights again question the natural link between people, state, and nation (Raj 2015) which emigrant states try to establish through the notion of diaspora. Diasporaas a unitary category therefore becomes unsettled (Ho 2011), and requires unpacking.

## Contextualizing emigration from India

Historical and structural context matter in the formation of the boundary line for (national) membership, such as citizenship (Yang 2000), and when creating diasporic identities (Raj 2015). I therefore provide an overview of India’s emigration history, which has been intertwined with the country’s experience of colonialism, state socialism and non-alignment, and economic liberalization (Raj 2015), in addition to the socioeconomic conditions that frame low-wage labor migration. According to official figures, approximately 32.1 million people of Indian origin live abroad. The Indian government categorizes them as NRIs (Non-Resident Indians, Indian nationals living outside India for over 183 days a year) (13.5 million) and People of Indian Origin (PIO) (first-generation, naturalized former Indian citizens and descendants of Indians up to the fourth generation) (18.6 million) (MEA 2022b). The Indian state has experienced a complex (e)migration trajectory, intertwined with the partition of British India and stretching back to its pre-independence era: a significant fraction of PIOs descend from indentured labor migration (Khadria 2006). Central to the partition of British India and the formation of its successor states, India and Pakistan, were religious/secular ideas of nationhood and belonging. Based on the logic of partition, Pakistan became the home of South Asian Muslims, making India the de facto haven for Hindus and other non-Muslims.^3^ Thus, tensions around religious-based selection criteria in definitions of who counts as belonging have been deeply intertwined with Indian state-building processes.

India’s post-independence emigration trajectory has broadly developed around three temporal-geographic poles. First, in the 1950s, Indians migrated predominantly to the United States, the United Kingdom, Canada, and Australia, where they often became naturalized (Xavier 2011). Indian migration to the GCC countries rapidly increased with the oil boom in the 1970s (Khadria 2006). Since the 1990s, Europe has increasingly been a target region, first for Indian knowledge workers and, in the last decade, also for low-wage workers (Sasikumar and Thimothy 2012). Although there are clear trends, linking high/low-wage migration to specific destination countries in general terms is problematic as, for example, migrants may have to take on lower-skilled employment in their destination context compared to their employment and qualifications at home (see Ho 2011).

Even in India, prospective low-wage emigrants experience fragmented access to citizenship rights, which I understand as a form of precarious citizenship (see Lori 2017). Their precarious status continues in the destination countries, particularly in the GCC countries, where they often experience major human and working rights abuses (Rajan, Varghese, and Jayakumar 2010). Central to their protracted precarity is ‘a lack of rights to work (at home) and a lack of rights at work (abroad)’ (Piper and Withers 2018, 565). Their employment in India is predominantly informal, creating pressure to migrate out of financial necessity (Piper and Withers 2018). As I will show in the next section, the Indian government defines a low-wage labor migrant as someone who left high school without a diploma, thus indicating their socioeconomic marginalization. Data on religious and caste composition are limited, but indicate an increase in migrants from marginalized groups since the early 2000s (see Kumar and Rajan 2014), working primarily in India’s informal economy (Mosse 2018). In the GCC countries, migrants on temporary contract migration schemes work predominantly in low-wage sectors, such as construction or domestic work (Fernandez 2021). Their residence visas are valid for three years, with no possibility of family reunification, permanent residence, or citizenship acquisition (Ali 2011). Migration to the GCC countries is often circular, leading to a ‘permanent temporariness’ (Piper and Withers 2018). The Kafala sponsorship system, prevailing in several GCC states, ties migrant workers to their employers and reinforces the vulnerability of migrant workers (see Fernandez 2021).

## India’s emigration and emigrant policies

The following analysis focuses on three examples of emigration and emigrant policies. I identify these examples as sites of substantive and symbolical identity-making activities, where the Indian state operationalizes the categories of low/high-skilled migration and redistributes social and cultural capital. 1) Emigration law: while the right to leave one’s country of nationality is a universal human right, the Indian state’s restrictions on emigration demonstrate that such bordering practices of exit are not natural but encompass constructions of subjectivities tied to the governance of belonging. 2) Voting rights and the OCI as citizenry rights: (political) rights are essential to defining membership of the democratic nation-state. 3) The Overseas Indian Day and its award as symbolic emigrant policies: both highlight the de-territorial dimension of membership construction, in how they function as networks and relate to place, and, importantly, are tied into the larger territorial-political project of establishing a Hindu-majoritarian state.

The analysis draws on interpretative approaches to study policies (Bacchi 2009; Stone 2012), that focus on understanding policies by examining the underlying beliefs, identity, and values embedded in them (Stone 2012). It studies together what the Indian state treats as two different sets of emigrants/citizens, and thereby highlights the absence of low-wage employed emigrants from its diaspora narrative. The analysis is thus informed by a contrapuntal reading – an approach to studying relationships of co-constitution in the production of texts between the metropolitan center and the subjugated groups, inspired by postcolonial IR scholars (e.g. Ho and McConnell 2022), who build on the work of Said (1993). One aim of contrapuntal analysis is ‘making visible the erasures and silences around concepts such as culture and identity, nation and memory’ (Chowdhry 2007, 102) and revealing the ‘mutually embedded histories (…) of the elite and subaltern’ (Chowdhry 2007, 105) by reading ‘the experience and histories of the privileged (…) against the histories of the dispossessed and marginalised’ (Chowdhry 2007, 106). I used primary sources – documents from the Indian government, information displayed on the MEA’s website – and newspaper articles as secondary sources. I analyzed these through a close reading of the categories by which policies are structured, such as their underpinning ideas and definitions regarding eligibility (inclusion) for policies.

### The Indian Emigration Act (1983) and differentiation in emigration policies

The first examples of boundary making are the Emigration Act of 1983 and its successor, the 2019 Emigration Draft Bill. With the Emigration Act, the Indian state established de facto two classes of passports and categorized its emigrants on the basis of education and therefore, implicitly, class. This differentiation in *emigration* policy has been decisive for all following *emigrant* policies, thus consolidating classed boundaries. It can be seen in their institutionalization from 1999 onwards, targeting emigrants and their descendants in countries of the Global North, but not emigrants residing in the GCC and other low-wage labor destination countries; it is also noticeable in the social protection policies, introduced in 2003, which target exclusively low-wage labor, female and student migrants.

The Emigration Act divides migrants into two categories: Emigration Check Required (ECR), and Emigration Check Not Required (ECNR). ECR status is assigned by means of a stamp in the passports of persons with a high school qualification below class 10, who intend to migrate to countries of the GCC region and to several other Middle Eastern and Southeast Asian countries.^4^ As illustrated later in the article, the policies of the Ministry of Overseas Indian Affairs (MOIA) and the Ministry of External Affairs (MEA), as well as their official documents, implicitly equate destination region and skill level.

Passports define their holders’ symbolic and material membership of a nation-state and express a relationship with fellow citizens (Bivand Erdal and Midtbøen 2021). The Indian passport’s value in providing resources for mobility depends on its holder’s educational level and emigration destination. Although ECR category migrants possess an Indian passport, the ECR category stamp implicitly turns it into a ‘second-class’ travel document, limiting and controlling mobility (rights). The duality established by the ECR and ECNR categories functions as an emigration control mechanism for GCC-bound migrants, while emigration is unregulated for migrants destined for Global North countries (see Kumar and Rajan 2014). Whereas the stance towards the latter emigrants resembles the ‘Nehruvian doctrine’ of non-interference dominant in India’s first post-independence decades (Lall 2003), the requirement for emigration clearance of people with ECR status reflects a discriminatory law (Rajan, Varghese, and Jayakumar 2010).

The differentiation in passports shows that low-wage labor emigrants are not full members and do not enjoy full human rights, such as the right to exit. Moreover, by providing some citizens with more rights than others, the emigration regulations create and re-enforce uneven social relations among citizens. In the case of the Emigration Act, specifically through the ECR stamped passport, the state regulates and creates differentiated belonging along classed boundaries.

The logic behind the ECR emigration regulations, as an inherently limited and hierarchal membership, becomes more evident in light of their colonial legacy and the history of India’s passport policy. With the official abolition of slavery in 1834, indentured workers from the British empire replaced enslaved people in labor-intensive plantation economies, including in Mauritius, the Caribbean, South America, and South Africa. Certification of identity was given ‘only for Indians of means, education and “respectability”’ (Singha 2013). (South Asian) indentured laborers did not receive passports, but instead, the British empire regulated and controlled their mobility, and that of formerly enslaved people, through emigration and immigration controls (Mongia 2018) and ‘coolie agreements’ (Singha 2013). These regulations also included the office of Protector of Emigrants, and a licensed recruitment regime, established in the 1860s (Rajan, Varghese, and Jayakumar 2010) to ensure that indentured workers emigrated voluntarily – and thereby were distinguishable from the unfree slave trade (Mongia 2018). The colonial state portrayed these regulations as exceptional, given the then general principle of free movement, and justified them by the ‘necessary ignorance’ (Mongia 2018, 16) of the colonized subjects and the existence of deceiving native recruiters (ibid.). A facilitation logic was behind the regulations, which aimed to ensure ‘free’ migration through state interventions (Mongia 2018). The 1983 Emigration Act is a continuation of the 1922 Indian Emigration Act; it essentially copied and re-established colonial emigration governance institutions (Kumar and Rajan 2014), such as the Protector General of Emigrants office, which administers ECR passport holders’ emigration clearances through field officers, registration with migrant recruitment agencies and their adherence to official recruitment regulations (Rajan, Varghese, and Jayakumar 2010). The independent Indian state retained the Emigration Act (1922) until the early 1980s, to severely restrict the emigration of low-wage Indians by granting very few emigration clearances (Agarwala 2022). Furthermore, Natarajan (2022) describes how, until 1967, the Indian state tried to curtail and prevent the emigration of ‘lower’ caste and class citizens, who were associated with indentured labor migrants, through a discretionary passport policy: it regarded the potential migrants as unfit representatives of the Indian state abroad. It thus made ‘the Indian passport a document of privilege, embodying the intersections of caste and class’ (Natarajan 2022, 3).

The Indian state justifies its dual emigration policy in terms of protecting ECR category migrants from exploitative or discriminatory labor conditions. More specifically, an application for emigration clearance can be rejected if the employment ‘is violative of norms of human dignity and decency’ (The Emigration Act 1983, 22(5b)) or if the potential migrant ‘will have to work in sub-standard working or living conditions’ (The Emigration Act 1983, 22(5c)). According to this reasoning, emigration control mechanisms are necessary to ensure the migration of low-wage employed workers as free subjects. However, controlling low-wage emigrants’ emigration reflects patriarchal norms, whereby the state classifies low-wage emigrants as needing protection and surveillance (see Shivakoti, Henderson, and Withers 2021). Furthermore, the mechanisms and regulations applicable to ECR-categorized migrants have not prevented cases of exploitation and abuse by recruitment agencies or employers, but rather have heightened their vulnerability. As a result, potential migrants use other means of travel, such as forged passports and visit visas, to avoid emigration controls. Consequently, these migrants are not recorded in the emigration register, making it administratively more difficult for the Indian state to intervene.

The Emigration Act (1983) should not be regarded solely as the outcome of institutional state incapacity (Sadiq and Tsourapas 2021). Rather, it reflects the relation between the Indian state and low-wage emigrants, defined by the latter’s precarious citizenship, which determines emigration regulations. An absence of stateness (see Slater and Kim 2015) and lack of access to rights often determine the life of a future GCC labor migrant *before* emigration. The rationales of difference, control, and surveillance, embedded in the claim for protection, still inform the Indian state’s normative understanding and its relation to low-wage emigrants (see Natarajan 2022).

The formation of the MOIA in 2004, the digitalization of the emigration clearance process (‘e-Migrate’) in 2015, and several other emigrant policies, described in the following sections, show that the Indian state has allocated resources to govern international migration but that the underlying rationales have not changed. Furthermore, in the summer of 2021, the MEA proposed introducing the Emigration Bill 2019 in parliament to replace the Emigration Act (1983) (MEA 2021b). Although the MEA plans to abolish the ECR/ECNR categories, the proposed law makes registration of persons who migrate through recruitment agencies – now re-named Human Resource Agencies – mandatory. In contrast, migrants who fall under the ECNR category can voluntarily register with an app, thus maintaining the distinction in the emigration process based on a migrant’s educational level and occupation.

The following sections show that the classed distinctions based on skill level and destination country set out in the Emigration Act (1983) prevail throughout India’s emigrant policies, including the OCI. The 2001 report of the High-Level Committee on the Indian Diaspora, commissioned by the MEA, first proposed a set of emigrant policies, which were subsequently implemented. In the following pages, I examine policies recommended in the MEA’s report, which are differentiated according to destination regions, along an ECR/ECNR divide. Policies for ENCR category migrants entail several ‘embracing’ strategies (Délano and Gamlen 2014), promoting a sense of belonging by deploying symbolic and substantial acts of inclusion, such as the extension of national membership through forms of transnational citizenship (see Raj 2015) and the production of a relationship of communication through the Overseas Indian Day. For ECR category migrants, the policies center on social protection provisions, aiming to decrease vulnerabilities due to economic and social risks and deprivation (see Kapur and Nangia 2015). Furthermore, the report initiated the establishment of the MOIA in 2004, which was merged again with the MEA in 2016 (*The Times of India*, 8 January 2016).

### Re-defining belonging by re-configuring (political) rights

In 2003, the Indian government introduced the OCI (Overseas Indian Citizenship) as an extension of the PIO (People of Indian Origin) card. OCI holders enjoy similar citizenry rights to Indian citizens and NRIs, except for political rights (Xavier 2011) (see Appendix Tables 2, 3, 4). The OCI is one example of how the Indian state has been re-defining the acquisition of Indian citizenship, from a birth-based principle to a descent-based principle. The OCI shows how the politics of belonging play out in both exclusionary and inclusionary ways and span the regulation of emigrants and immigrants, with boundaries to membership which simultaneously expand and contract. It was tied to the Citizenship Amendment Act of 2003, which introduced a religious-based exception for acquisition of citizenship at birth, by excluding descendants of an undocumented migrant parent from acquiring Indian citizenship when born on Indian soil and therefore implicitly excluding children of Bangladeshi (Muslim) parents. New regulations introduced in 2021 mean that OCI card holders now have to have a special permit to undertake research, journalism, *Tabligh*, and missionary activities, for employment with India-based, foreign diplomatic missions or foreign government organizations, and for visits to any areas or places designated as protected/restricted/prohibited space. These restrictions can be viewed as attempts to suppress ‘anti-nationalist’ reporting and political dissent against the Bharatiya Janata Party-led government more generally (see Sharma 2021).

The OCI, which at first glance seems to be an inclusive policy seeking to broaden membership of the Indian nation, was initially also a site of exclusionary, classed boundary making, intended to exclude specific fractions of PIOs. Until its revision in 2005, only PIOs with citizenship of a limited number of European, Australasian, and North American countries were eligible to obtain the OCI, thus leaving out the substantial populations descended from pre-independence indentured migrants living in Africa, the Caribbean, and South America (Xavier 2011). Since 2005 all PIOs, except Pakistani or Bangladeshi nationals, have been entitled to the OCI (Xavier 2011). The PIO and OCI schemes ran in parallel until 2015, when the PIO scheme merged with the OCI. Since then, persons with Indian ancestry up to the fourth generation and spouses of OCI holders can acquire the OCI (MEA 2022a), thus enlarging the eligibility criteria.

Although political rights – standing for election and voting – have been the one clear distinction between the OCI and full Indian citizenship, these rights are often nominal, rather than actual. India granted NRIs voting rights only in 2010, six years after introducing the OCI. Moreover, unlike for the OCI card, it was not the High-Level Committee that proposed the extension of voting rights to NRIs, but the Indian National Congress-led parliament, which passed the bill to ‘allow the NRIs to participate in the democratic process to boost the two-way engagement further’ (*The Economic Times*, 25 November 2010). Nonetheless, for the last eleven years, votes have to be cast in person in the voter’s constituency, a requirement that excludes persons who cannot (afford to) return to India to vote. This regulation leads to the de facto disenfranchisement of international low-wage migrants, who often return to India only every few years, making it unlikely for them to participate in elections.

However, it is not uncommon for political parties of major emigrant states, such as Kerala, to fly in voters in chartered planes, ahead of general and regional elections (Nijeesh 2019). Moreover, as social provision is associated with state governments and not union state programs (Tillin, Deshpande, and Kailash 2015), states have a stronger incentive to actively engage with the emigrant populations. Consequently, the sub-national citizenship of low-wage labor emigrants determines their substantive political rights, echoing what Yuval-Davis (1999) describes as multi-layered citizenship, where citizenship encompasses belonging and rights beyond the level of the nation-state, namely relating to sub-national, religious, and ethnic political communities.

In August 2018, the Indian parliament’s Lower House passed a bill proposing to grant proxy voting rights to NRIs. The bill did not pass the Upper House on the first attempt, but in 2020, the Election Commission approached the Indian government and proposed to grant postal voting rights to NRIs. With a view to the state elections, the Election Commission and the MEA planned to introduce postal voting for NRIs as a pilot in the US, Canada, New Zealand, Japan, Australia, Germany, France, and South Africa. The GCC countries were not considered for the pilot: the MEA expressed reservations ‘over seeking permission in non-democratic nations to facilitate postal voting for Indian citizens living there’ (Chopra and Roy 2020). Scholarship suggests that the current Bharatiya Janata Party-led government would benefit from the votes of NRIs residing in the former countries (Jayal 2019; see, e.g.; Wellman 2021 for dynamics of strategic enfranchisement in sub-Saharan Africa), whereas large numbers of NRIs in the GCC countries originate from South Indian states where the party traditionally does not hold majorities; the outcome of external voting would therefore be unpredictable. Theoretically, the extension of postal voting rights would mean a shift towards realizing substantive political rights for all NRIs, but considering the pilot schemes’ target countries, the Indian state is leaving out the majority of low-wage labor emigrants.

### Symbolic boundaries: constructing belonging around professional success and Hindu nationalism

#### Pravasi Bhartiya Divas

One of the three initiatives stemming from the High-Level Committee report was the creation of Overseas Indian Day (‘Pravasi Bhartiya Divas’ (PBD)), in 2003. Promoted as a ‘flagship event’ (MEA 2022c), the aim of this day is to forge a connection between India and NRIs and OCIs residing abroad, providing an opportunity to network and exchange knowledge. It takes place once every two years ‘to strengthen the engagement of the overseas Indian community with the Government of India and reconnect them with their roots’ (MEA 2022c) and to ‘mark the contribution of Overseas Indian community in the development of India’ (MEA 2022c).

The event addresses the ‘overseas diaspora’ (MEA 2022c) and ‘overseas Indian community’ (ibid.); this is not an invitation to all Indians living abroad, but the MEA envisages the participation of ‘overseas diaspora experts, policymakers, and stakeholders’ (MEA 2022c). They are supposed to ‘bring their knowledge, expertise, and skills on a common platform’ (ibid.), ‘to share their experiences in various fields’ (ibid.). The Indian overseas diaspora is thus constructed around a notion of professional success, equated with occupation and skills and ‘participation in globalized networks’ (Dickinson and Bailey 2007, 765), implicitly excluding ECR category migrants as a target audience.

The PBD event is further class-differentiated in its organizational and spatial set-up, as the following analysis of the list of speakers, topics, and side events reveals (see MEA 2022c). The PBD is structured around topical sessions.^5^ Several sessions deal with destination regions, including the GCC countries. Referred to as ‘NRIs in the Gulf’ or ‘Indians in the Gulf’, the GCC countries have been a topical feature at each PBD event, demonstrating the region’s importance for India. The GCC-residing Indian speakers are Indian middle-class migrants: school principals, business directors, doctors, engineers, journalists, and cultural conveners. Low-wage migrants are not present as speakers and are excluded from representing and lobbying for their interests. Instead, a limited number of representatives of Indian community organizations, such as the Pravasi Rehabilitation Center, Saudi Arabia (PBD 2008, 2009) and the Indian Community Welfare Committee, Dubai (PBD 2006), raise issues possibly affecting low-wage migrants.^6^ In 2019, a whole panel was dedicated to the ‘Indian community organizations working for Indian nationals in distressed situations’ (MEA n.d.), with representatives residing in GCC countries and Australia, New Zealand, the UK, and the US; it thus became a platform where middle/upper-class Indian migrants brokered rights claim-making on behalf of low-wage migrants. Other sessions discussed development as a philanthropic activity, without acknowledging the (economic) contributions of GCC-bound low-wage migrants (see, for example, Jain 2016). Speakers on behalf of low-wage migrants in the GCC countries, such as the Indian Community Welfare Committee Dubai, point out that ‘the NRI Day has become more of a platform for Indian businessmen to conduct their networking’ (Menon 2007). As a result, they were no longer interested in participating in the PBD, voicing criticism that ‘Indians living in the Gulf are not given any special treatment because although we live outside India we are still Indians. This is because we do not have the option to take dual nationality’ (Menon 2007). These expressions of resentment echo the findings of Mani and Varadarajan (2005), who describe how, even at the first PBD in 2003, delegates showed skepticism and resistance to the Indian state’s nationalist, universalizing narrative of a homogenous diaspora.

More recently, the venue and date of the PBD indicate a shift away from Gandhi-referred nationalism (see Mani and Varadarajan 2005) to a majoritarian Hindu notion of Indian nationalism, in which the claim to belong through participation is differentiated not only along classed but also along religious lines. In 2019, the year of the national elections, the date of the PBD was postponed by two weeks; it then took place days before Indian Republic Day and at the same time as the Hindu religious festival Kumbh Mela, the most significant Hindu pilgrimage event globally. The MEA advertised participation in the PBD as a package, combined with visits to the Kumbh Mela and Republic Day (MEA n.d.). In tandem with the state of Uttar Pradesh, the MEA organized the event in Varanasi. The city is a sacred Hindu site – the holiest city for Hindus – and is the electoral constituency of Narendra Modi, who was running for Prime Minister for the Bharatiya Janata Party in the upcoming 2019 general elections.

Although the PBD has taken place in different locations since its inauguration, the choice of Varanasi as a venue and of Modi’s electoral constituency fits well with what Deshpande (1995) and Oza (2007) describe as the Hindu Right’s spatial strategies to create a Hindu nation-state. These spatial strategies aim at reclaiming public space (neighborhoods), sacred sites (shrines, mosques), and imagined space (the nation) to turn them into Hindu spaces (Deshpande 1995; Oza 2007). Constructions of (spatial) belonging categories are the ideological underpinning of the Hindu Right’s spatial strategies, rooted in ‘a discourse of legitimacy whereby Hindus have the sole rightful claim to the nation’ (Oza 2007, 154). Hindus are considered to be the original inhabitants of India. In contrast, Muslims are said not to belong to the Indian nation and, in reference to the alleged violent expansion of Islam in South Asia, have been seen as imperialists subjugating Hindus (Oza 2007). The spatial belonging and segregation narrative has been complicated by intertwined material, symbolic, linguistic, and cultural spaces that defy clear demarcations between ‘us’ and ‘them’ (Oza 2007). Attempts to spatially purify sacred sites along a Hindu-Muslim divide, by violent means, such as the Ram temple movement/the destruction of the mosque in Ayodhya (see Oza 2007), have been part of this ‘strategic deployment of essentialism’ (Deshpande 1995, 3223).

As part of the Kumbh Mela, processions and pilgrimages are space-claiming and mobilization strategies (Jaffrelot 2009; Deshpande 1995). Hindu nationalists have re-interpreted and instrumentalized the traditional Hindu pilgrimage since the 1980s, through pan-Indian pilgrimages. By holding the PBD in Varanasi and linking it to the Kumbh Mela, performance and claims to belong through participation in the PBD are subsumed into heritage-making and space-claiming processes, entangled with Hindu nationalism. This creates inherent outsiders who cannot or do not want to perform religious nationalism. Nevertheless, pilgrimages have an egalitarian quality that produces a ‘temporal obliteration of social cleavages – particularly caste divisions – allowing the emergence of a sense of group belonging around a particular identity’ (Jaffrelot 2009). They incorporate and repress social differences, such as caste, class, and gender, but enhance nationality through ethnicization processes (see Natrajan 2021), in which ethnic identity identifies with religion-as-culture (Brosius 2005).

#### The Pravasi Bharatiya Samman award

On the Overseas Indian Day, ‘eminent cultural, literary, and sports personalities’ (MEA 2001) are honored with the *Pravasi Bharatiya Samman Award*, to recognize their contributions to various fields in India and abroad (ibid.). Like the PBD, the award ceremony serves to establish a sense of Indian membership using symbolic acts, producing a relationship of communication (see Waterbury 2010).

On the nomination form, the nominee has to demonstrate belonging by providing proof of familial bonds to India: the form asks for the names of the nominee’s ancestor who migrated from India, their emigration year, last known address, and their closest relative in India, before asking for justification of the nomination (see MEA 2020). Indian migrants in the GCC countries have received numerous awards (see Appendix Table 2). Persons or organizations are eligible if they have made ‘a significant contribution’ to one of the following fields: ‘Better understanding abroad of India; Support to India’s causes and concerns in a tangible way; Building closer links between India, the overseas Indian community, and their country of residence; Social and humanitarian causes in India or abroad; Welfare of the local Indian community; Philanthropic and charitable work’ (MEA 2020). The award’s eligibility criteria are already classed: many low-wage migrants live spatially segregated (Mohammad and Sidaway 2016) in so-called work camps, often outside the main cities or in rural areas, and work with other migrants, so their contact with the local population is minimal. In addition, many domestic workers never leave their employer’s house (Gamburd 2009). One exception to this is the category of persons with ‘Eminence in skills which has enhanced India’s prestige in that country (for non-professional workers)’ (MEA 2020). The last criterion refers to artisans and artists who are highly skilled in their profession but do not have a high school or university degree. Among all 239 awardees (2003–2015; 2017), no artisan or artist has ever received the award (see MEA 2020).

## Conclusion

Building on the concept of boundary work and critical approaches to the study of diasporas, this article examined how the Indian state creates lines and categories of belonging through differentiated emigration and emigrant policies and practices, such as externalized citizenship-like rights and associated privileges. It compared and contrasted India’s policy responses directed at citizens living abroad and former citizens and their descendants, analyzing three examples of emigration and emigrant policies: (1) emigration law, (2) political rights, and (3) symbolic policies. The article showed that the Indian state organizes these policies and practices around classifications, such as class and religion, which create hierarchical, multi-layered, and intersectional dimensions of belonging and membership. The independent Indian state has tried to accommodate its population’s social diversity by means of its secular constitution, its initial conceptualization of citizenship in terms of a birth-based principle (Indian Citizenship Act of 1955), specific institutions such as the National Commission for Minorities, and a democratic, competitive political process (Mitra 2008). However, the uneven distribution of legal and substantive citizenship rights, negatively affecting marginalized individuals and communities, also becomes apparent in the ways in which emigrant and emigration policies are conceptualized and practiced.

I traced these instances of differentiation back to institutional legacies of colonial rule, which created hierarchies between colonizers and colonized, and within colonized populations, based on mobility/emigration and, more generally, socioeconomic cleavages. The Indian emigration and emigrant policies are another instance of how colonialism configures mobility regulations and exclusionary/inclusionary policies (see Mayblin and Turner 2021). Therefore, this article connects to the broader debates about the hierarchization of human worth (see, for example, Welfens 2021) – a relevant issue beyond postcolonial societies.

By focusing on the domestic contexts, the research adds to the existing literature on emigrant policy differentiation, which has predominantly used bilateral and international explanations, such as geopolitical, economic, and foreign policy interests, to understand the target groups of policies deployed by origin states. In addition, it contributes to the scholarship which questions India’s universal narrative and dominant analyses of India’s emigrant incorporation as an unambiguous success story (e.g. Naujoks 2010), by pointing to the inequalities and exclusions experienced by low-wage labor emigrants. While this article highlights emigrant and emigration policies as sites of class reproduction, and responds to calls for more attention to be paid to the role played by class in migration policy (Bonjour and Chauvin 2018), further ethnographic work needs to explicitly demonstrate how caste-based differentiation and exclusion intersect with class as an underlying category of difference.

This article implies that emigrants’ social positions, as outcomes of emigrant policies, cannot be read solely with regard to the Indian federal state, but need to be viewed in terms of their inclusion/exclusion on multiple – such as subnational and international – scales. Low-wage migrants from Kerala, for example, enjoy more privileges and rights than their colleagues from Bihar, because of interventions by the government of the former state. Lastly, the in/exclusionary boundaries in the status and rights dimension of India’s emigrant policies reflect broader re-configurations of Indian citizenship, in the last two decades, towards a Hindu-nationalist majoritarian and exclusionary concept of citizenship. This re-configuration has most recently been exemplified by a double process. Firstly, 2019 saw the adoption of the Citizenship (Amendment) Act, through which the Indian state no longer considers undocumented immigrants from Afghanistan, Bangladesh, and Pakistan as illegal if they belong to religious minorities, thus excluding Muslims, and grants them fast-tracked access to Indian citizenship (see Jayal 2019); secondly, Muslims have faced illegalization and loss of citizenship in Assam through application of the National Register of Citizens, with the prospect of nation-wide implementation (Punathil 2022).

## Notes

1. I use the term low-wage migrant so as to not reproduce state-informed categorical hierarchies. Referring to categories applied by the Indian state, I use the terms low-skilled/high-skilled migrants.

2. The Indian state only collects data on persons who emigrate for low-wage employment, so no data on the occupations of Indian migrants in particular destination countries is available. Khadria (2006) estimates that about 70% of Indian migrants to the GCC countries are in low-wage employment.

3. About 35 million Muslims remained in India, i.e. approximately 10% of the total population in 1947 (Tan and Kudaisya 2000).

4. The 18 ECR countries are the Kingdom of Saudi Arabia (KSA), the United Arab Emirates (UAE), Qatar, Oman, Kuwait, Bahrain, Libya, Jordan, Yemen, Sudan, South Sudan, Afghanistan, Indonesia, Syria, Lebanon, Thailand, Iraq and Malaysia (MEA 2016).

5. The detailed list of speakers and of the topics of sessions is available for the years 2003–2013 and 2019 (see MEA 2022c). Other topics can be categorized as: business, finance and trade; public policies; media, entertainment, and culture; development and philanthropy; engagement with Indian state governments.

6. Four representatives in total for the PBDs (2003–2013) (MEA 2022c).

## Appendix

**Table 1.** Overview of social protection schemes (see MEA 2021a).

	Social Assistance	Labor Market Programs	Social Insurance
Pre-departure	**Overseas Workers Resource Centre (OWRC)** (2004) in Delhi, Chennai, Lucknow, Kochi and Hyderabad: Grievance registration Tracking of grievance Assistance for online Migration Clearance (e-Migrate) Toll-free 24 × 7 helpline support in various Indian languages	One-day pre-departure training (2018)	
Destination Country	**Indian Community Welfare Fund (Emergency Fund)** (2009) Assisting in distress situations Support for Community Welfare activities Improvement in Consular services **Pravasi Bharatiya Sahayata Kendra (PBSK) (2010)** in Dubai and Sharjah (UAE), Riyadh and Jeddah (Kingdom of Saudi Arabia); Kuala Lumpur (Malaysia) 24/7 helpline walk-in counter in Dubai and Sharjah, legal, psychological and financial counselling awareness campaigns in workers’ accommodation sites **Online Grievances complaint form (MADAD)** against Indian embassy services (2015)		**Compulsory Insurance Scheme** Pravasi Bharatiya Bima Yojana (PBBY)) (2003): Cover sum accidental death/permanent disability One-way ticket for rehabilitation in case of death or disability Medical treatment for migrant and their families in case of illness Maternity expenses Legal expenses related to employment abroad
Return			Voluntary Pension scheme (discontinued in 2017): Mahatma Gandhi Pravasi Suraksha Yojana (MGPSY) (2012)

**Table 2.** Symbolic policies (see MEA 2020, 2022a, 2022c).

		Citizenship status/Emigration category	
Policy	Non-Resident Indians (Indian citizenship): **Emigration Check Not Required** (ECNR)	Non-Resident Indians (Indian citizenship): **Emigration Check Required** (ECR)	**Overseas Citizenship of India (OCI)** (Citizenship of other countries) (2005)
Pravasi Bharatiya Samman Award (2003–2015) 179 Awards in total	4 (Bahrain) 2 (Kuwait) 6 (Oman) 3 (Qatar) 5 (Saudi Arabia) 11 (UAE)	0	32 (USA) 12 (UK) 5 (Canada) 4 (Australia)
Additional benefits			At Parity with NRIs for Inter-country child adoption Admittance into national parks, museums, national heritage, etc.

**Table 3.** Emigration regulations (see MEA 2016).

		Citizenship status/Emigration category	
Policy	Non-Resident Indians (Indian citizenship): **Emigration Check Not Required** (ECNR)	Non-Resident Indians (Indian citizenship): **Emigration Check Required** (ECR)	**Overseas Citizenship of India (OCI)** (Citizenship of other countries) (2005)
Emigration	No restrictions	Emigration clearance needed	No restrictions

**Table 4.** Citizenry rights (see MEA 2022a; Xavier 2011).

		Citizenship status/Emigration category	
Policy	Non-Resident Indians (Indian citizenship): **Emigration Check Not Required** (ECNR) (1983)	Non-Resident Indians (Indian citizenship): **Emigration Check Required** (ECR) (1983)	**Overseas Citizenship of India (OCI)** (Citizenship of other countries) (2005)
SocialProtection Policies (See Appendix Table 1) (Social rights)	Only Students (MADAD registration; Indian Community Welfare Fund) and Women (Indian Community Welfare Fund)	All	No
Economic rights	Full economic rights	Full economic rights	Full economic rights, but no acquisition of agricultural property
Political rights	Since 2010 voting rights, only in person in the home constituency	Since 2010 voting rights, only in person in the home constituency	No political rights: no right to vote, to stand for elections, or admission to public service
Work permit	Full access to labor market	Full access to labor market	Eligible for appointment as Teaching faculty in IITs, NITs, IIMs, IISERs, IISc, Central Universities, AIIMS Doctor, dentist, pharmacist, nurse, advocate, chartered account, architect In other sectors, the same rights and privileges as a foreigner
Educational rights	Access to all Higher Educational Institutions/Reserved seats for NRIs Scholarship program for Diaspora Children (2006)	Access to all Higher Educational Institutions/Reserved seats for NRIs Scholarship program for Diaspora Children (since 2017 reservation)	Access to all Higher Educational Institutions/at parity with NRIs for reserved seats Scholarship program for Diaspora Children (2006)
